# Hybrid Tomo‐Helical and Tomo‐Direct radiotherapy for localized prostate cancer

**DOI:** 10.1002/acm2.13406

**Published:** 2021-09-08

**Authors:** Sibel Karaca, Timur Koca, İsmail Hakkı Sarpün, Nina Tunçel, Aylin Fidan Korcum Şahin

**Affiliations:** ^1^ Department of Radiation Oncology Faculty of Medicine Akdeniz University Antalya Turkey

**Keywords:** hybrid radiotherapy, localized prostate cancer, Tomo‐Direct, Tomo‐Helical

## Abstract

**Purpose:**

The aim of the study is to present a new planning approach to provide better planning target volume (PTV) coverage and reduce bladder and rectum dose with hybrid Tomo‐Helical (TH)/Tomo‐Direct (TD) radiotherapy (RT) for localized prostate cancer (LPC).

**Methods:**

Twenty‐five LPC patients were included in this retrospective study. TH plans, TD plans, and hybrid TH/TD plans were created. Lateral beams were used for the hybrid TD plan and the prescribed dose was 70 Gy in 28 fractions (hybrid plans were combined 45 Gy/ 18 fxs for TH and 25 Gy/10 fxs for TD). Doses of PTV (D2%, D98%, D50%, homogeneity index (HI), conformity index (CI), coverage) and organs at risk (OARs) (V50%, V35%, V25%, V5%, and V95%) were analyzed. The Wilcoxon signed‐rank test was used to analyze the difference in dosimetric parameters. *p‐*Value < 0.05 was considered statistically significant.

**Results:**

TH plans showed better CI, and target coverage (*p* < 0.01) than TD and hybrid plans in all patient plan evaluations. However, TD plans D2%, D98%, and D50% doses were better than TH and hybrid plans. The HI values were similar between the three plans. Significant reductions in bladder and rectum V50%, V35%, and V25% doses (*p* < 0.001) were observed with hybrid plans compared to TH and TD. Penile bulb V95% and bowel V5% doses were better in the hybrid plans. Left and right femoral head V5% doses were higher in the hybrid plan compared to others (*p* < 0.001).

**Conclusion:**

Concurrently hybrid TH/TD RT plan can be a good option to reduce the doses of the rectum and bladder in the RT of LPC.

## 1 INTRODUCTION

Prostate cancer is one of the most common types of cancer among men and primary radiotherapy (RT) is an established treatment option for patients with localized prostate cancer (LPC).^1^, ^2^ Nowadays, LPC is treated with advances in RT such as intensity‐modulated RT (IMRT) and image guide RT (IGRT) have helped to reduce toxicity.^3^, ^4^, ^5^, ^6^ IMRT method allows dose escalation with high conformity and high dose to the prostate while sparing organs at risk (OAR) (rectum, bladder, bowel, and femoral heads).^7^, ^8^ Studies on prostate cancer have shown that high‐dose RT applications have significant contributions to treatment outcomes.^9^, ^10^ However, the hypofraction program recently has been accepted as an external beam RT strategy in prostate cancer.^11^ High‐dose moderate hypofractionation RT is thought to reduce the effect of tumor cell repopulation^12^ and improve treatment outcomes in the RT of LPC.^13^, ^14^, ^15^, ^16^, ^17^

Tomotherapy (Accuray Inc.) is an RT device with IGRT capability by taking daily Megavoltage Computed Tomography scan before treatment.^18^ Tomotherapy has different version treatment devices and one of them is TomoHDA. TomoHDA treatment system was produced after TomoTherapy‐Hi‐Art and TomoHD technologies. The first treatment with TomoHDA was performed at Center Oscar Lambret in Lille, France in 2013. TomoHDA system offers the user the chance to treat patients in two different modes. Tomo‐Helical (TH) mode provides a continuous 360° beam that may result in optimal dose distribution and dose conformity.^19^ The Tomo‐Direct (TD) mode is a non‐rotational treatment option of the Tomotherapy platform and provides treatment at predetermined angles with a fixed gantry.^20^, ^21^ TomoHDA provides faster and better dose distribution with the new technology IDMS (integrated data management system) with *Precision* Planning System.^22^

In this study, we aimed to develop a new planning approach by creating simultaneous hybrid TH/TD treatment plans for LPC patients undergoing high‐dose RT with TomoHDA. We intended to reduce the doses of OARs such as bladder and rectum by increasing target dose homogeneity with the hybrid technique.

## 2 MATERIALS AND METHOD

### 2.1 Patient selection and contouring

In this study, we used image data of 25 patients with a diagnosis of LPC. The patients who were treated for LPC using Elekta Synergy Platform linear accelerator and Tomotherapy HDA between June 2018 and September 2020, were retrospectively analyzed. The patients were instructed to drink 500–750 ml of water 45 min before the CT scan (as our clinical protocol). Patients were asked to empty their bladder before treatment and were provided to be treated with an empty rectum. Patients were placed in the head‐first supine position and 2.5 mm slice thickness computed tomography (CT scan GE‐Light Speed 64 Discovery RT 16 Slice; GE) was performed on the T‐board in the supine arm up position. Magnetic resonance images were performed by a 3T whole‐body system (Spectra; Siemens‐Healthcare).

Radiation oncologists contoured all targets and OARs of LPC patients. The whole prostate contoured as clinical target volume and planning target volume (PTV) generated by clinical target volume plus 5 mm posteriorly and 10 mm in other directions. Contouring of PTV and OARs were performed by using a CT scan with a 2.5 mm slice thickness and magnetic resonance images fusion. Rectum, bladder, bowel, femoral heads, and penile bulb were contoured as OARs. Rectum was contoured from ischial tuberosities to rectosigmoid flexure and femoral heads inferiorly to the bottom of ischial tuberosities. Urinary bladder contoured from its base to the dome and penile bulb contoured immediately inferior to the genitourinary diaphragm.^23^

### 2.2 Treatment planning

TH, TD, and hybrid TH/TD plans were created in the Tomotherapy HDA IDMS Precision Planning System (version 2.0.1.1[5]; Accuray Inc.) for each patient. All plans were normalized to cover 95% of the PTV, and 70 Gy delivered at Gy 2.5 Gy/fraction in 28 fractions.^24^ Various parameters can be used in the tomotherapy planning station optimization panel. One of the optimization parameters is the *importance* and the other one is the *penalty*. Critical organs are listed in order of priority. For the plans to be comparable, the same parameters (priority, importance, maximum dose penalties, and minimum dose penalties) were determined in both TH and TD plans (Table 1). *A fine* calculation grid (1.95 × 1.95 mm)was used for the final calculation process. Optimization resolution was selected as *low*. Each plan calculated the final dose after 200 iterations.

**TABLE 1:** Planning priority, importance, maximum (max) dose penalty, minimum (min) dose penalty, max dose, dose–volume histogram (DVH) volume (%), and DVH dose (Gy) for planning target volume (PTV) and organs at risk (OARs)

**Structures**	**Overlap priority**	**Importance**	**Max dose (Gy)**	**Max dose penalty**	**DVH vol (%)**	**DVH dose (Gy)**	**Min dose (Gy)**	**Min dose penalty**
PTV	1	100	70	100	95	70	70	100
OARs								
Bladder	1 (1 = highest priority)	1	1	1	1	1	1	1
Rectum	2	1	1	1	1	1	1	1
Bowel	3	1	1	1	1	1	1	1
Right femural head	4	1	1	1	1	1	1	1
Left femural head	5	1	1	1	1	1	1	1
Penile bulb	6	1	1	1	1	1	1	1

### 2.3 Tomo‐Helical, Tomo‐Direct, and hybrid planning

The TD IMRT plans were generated with dynamic jaw mode. The field width, pitch, and modulation factor values were 2.5 cm, 0.251, and 2.0, respectively. Treatment plans were generated using a seven beams arrangement with gantry angles of 0°, 52°, 103°, 154°, 205°, 256°, and 308°. The prescribed dose was 70 Gy in 28 fractions.

The TH IMRT plans were generated with helical dynamic jaw mode The field width, pitch, and modulation factor values were 2.5 cm, 0.314, and 2.0, respectively. The prescribed dose was 70 Gy in 28 fractions.

The hybrid tomotherapy plans were a combination of TH and TD plans. HD planning dose provided approximately two‐thirds of the total dose. TD planning dose provided approximately one‐third of the total dose. 25 Gy delivered at 2.5 Gy/fraction in 10 fractions. Seven lateral beams were used with gantry angles of 77°, 86°, 101°, 110°, 250°, 275°, and 300°. The prescribed dose was 45 Gy in 15 fractions and sum plans of TH/TD plans were determined.

### 2.4 Dosimetric evaluation

Plan evaluations were based on dose–volume histogram (DVH) analysis. To evaluate the dose distribution of the target, parameters were calculated for PTVs: the absolute dose received by the 2% (D2%), the absolute dose received by the 50% (D50%), the absolute dose received by 98% (D98%; ICRU 83).^25^

Conformity index (CI) is the ratio of total tissue volume that receives the prescription isodose or more to the target volume that receives the prescription isodose.^26^

(1) $$\mathrm{CI}=\frac{V_{\mathrm{RI}}}{\mathrm{TV}}$$

where *V*_RI_ is the volume covered by the prescribed dose and TV is the target volume.

Homogeneity index (HI) is the ratio of the maximum dose to the prescription dose.^26^

(2) $$\mathrm{HI}\;=\frac{D\max}{Dp}\;\;\;\;$$

where *D*_max_ is the maximum dose of the target volume and *D*_p_ is the prescription dose of the target volume.

Coverage is the volume of the tumor that receives the prescription dose divided by the total volume. For the bladder and rectum, the DVH points of V50(%), V35(%), V25(%), V20(%), and V15(%) were examined. Also, the doses of femoral heads V5(%), bowel V5(%), and penile bulb V95(%) were evaluated.

### 2.5 Statistical analyses

Statistical analyses were performed with SPSS 18.0 (Statistical Package for the Social Sciences; SPSS Inc.). The Wilcoxon signed‐rank test was used to compare the dose parameters of the TD and TH plans. All *p‐*values below 0.05 were considered statistically significant.

## 3 RESULTS

**FIGURE 1:**
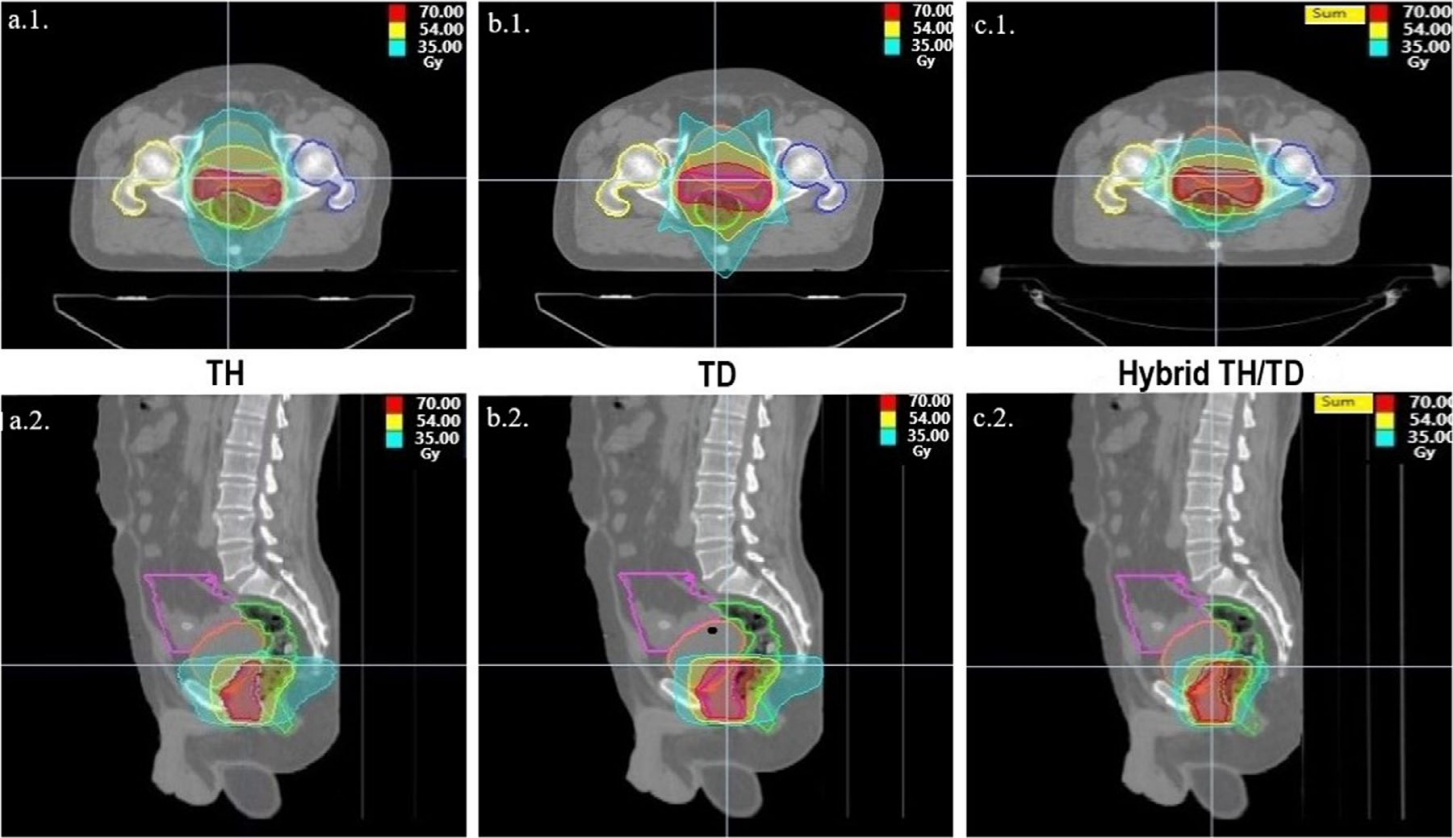
Axial and sagittal dose distribution of localized prostate cancer patients. (a1 and a2) Tomo‐Helical (TH) plan, (b1and b2) Tomo‐Direct (TD) plan, and (c1 and c2) hybrid TH/TD plan

In this study, we investigated whether there was a statistically significant difference in dosimetric values between the TH‐TD, TH‐hybrid TH/TD, and TD‐hybrid TH/TD plans. Tables 2, 3, 4 summarize the DVH parameters for PTV and OARs of the three plans in 25 patients (total 75 plans). The mean volume of PTV was 146.559 cc (range: 70.51–318.48 cc). For PTV, the D2%, D50%, and D98% values in TD were significantly better than other plans (*p* < 0.01). HT provided for better dose CI (*p* < 0.05) and coverage (%) than others. The HI values were similar between the three plans (*p* > 0.05). The mean volume of the bladder was 265.515cc (range: 72.53–494.25 cc), the rectum was 83.914 ± 46.148 (cc) (range: 35.32–220.29 cc), and the bowel was 1375.541 (range: 720.13–3258 cc). The hybrid TH/TD plans provided statistically significant sparing for both the bladder and rectum at all V50(%), V35(%), and V25(%) values (*p* < 0.01). At the same rectum and bladder, the V15(%) value of the hybrid TH/TD plan is more advantageous than TH and TD plans. The right femoral head and left femoral head V5% doses were statistically significantly higher in the hybrid TH/TD plan compared to TH and TD (*p* < 0.001). We found no significant difference for the bowel V5% and penile bulb V95% among the three plans (*p* > 0.05). But bowel V5% and penile bulb V95% hybrid TH/TD plan value was the lowest value when compared with other plans. A comparison of axial and sagittal dose distribution of LPC patients for TH, TD, and hybrid TH/TD plan is shown in Figure 1.

**TABLE 2:** Dose–volume histogram (DVH) parameters (mean ± standard deviation) for planning target volume (PTV) and organs at risk (OARs) with *p*‐values for comparison of Tomo‐Helical (TH) and Tomo‐Direct (TD)

**Parameters**	**Unit**	**Tomo‐Helical**	**Tomo‐Direct**	***p‐*Value Tomo‐H versus Tomo‐D**
PTV				
PTV Vol	146.559 ± 51.446 (cc)			
PTV D2%	Gy	71.385 ± 0.372	71.111 ± 0.334	<0.001
PTV D98%	Gy	70.312 ± 0.190	70.276 ± 0.124	0.037
PTV D50%	Gy	70.885 ± 0.311	70.742 ± 0.283	0.003
CI		1.400 ± 0.101	1.413 ± 0.138	0.047
HI		1.032 ± 0.006	1.149 ± 0.602	0.430
Coverage(%)		99.501 ± 0.423	99.332 ± 0.586	0.050
Bladder				
Bladder Vol	265.515 ± 120.05 (cc)			
V50(%)	Gy	25.600 ± 21.092	26.496 ± 21.191	<0.001
V35(%)	Gy	39.305 ± 21.191	40.219 ± 21.417	<0.001
V25(%)	Gy	49.403 ± 20.534	50.018 ± 20.367	0.010
V15(%)	Gy	60.884 ± 15.169	59.056 ± 17.823	0.221
Rectum				
Rectum Vol	83.914 ± 46.148 (cc)			
V50(%)	Gy	51.301 ± 9.909	51.170 ± 11.609	0.069
V35(%)	Gy	61.184 ± 6.305	61.529 ± 6.569	0.333
V25(%)	Gy	65.897 ± 4.271	65.855 ± 5.326	0.882
V15(%)	Gy	69.475 ± 1.772	69.081 ± 2.724	0.033
R Femur				
V5(%)	Gy	25.830 ± 4.834	26.180 ± 6.51	0.326
L Femur				
V5(%)	Gy	25.818 ± 5.716	27.148 ± 7.091	0.001
Penil Bulb				
V95(%)	Gy	45.788 ± 21.246	45.457 ± 20.924	0.667
Bowel				
Bowel Vol	1375.541 ± 1037.756 (cc)			
V5(%)	Gy	2.598 ± 1.746	2.66 ± 1.983	0.12

Abbreviations: CI, conformity index; Dxx(%), dose incident on xx% structure volume; Gy, gray; HI, homogeneity index; L femur, left femoral head; R Femur, right femoral head; Vol, volume; Vxx(%)Gy, % volume of structure receiving a dose of xx Gy.

**TABLE 3:** Dose–volume histogram (DVH) parameters (mean ± standard deviation) for planning target volume (PTV) and organs at risk (OARs) with *p*‐values for comparison of Tomo‐Helical (TH) and hybrid plans

**Parameters**	**Unit**	**Tomo‐Helical**	**Hybrid (45 Gy/25 Gy)**	***p‐*Value**
PTV				
PTV Vol	146.559 ± 51.446 (cc)			
PTV D2%	Gy	71.385 ± 0.372	71.188 ± 0.767	0.259
PTV D98%	Gy	70.312 ± 0.190	70.506 ± 0.394	0.002
PTV D50%	Gy	70.885 ± 0.311	70.971 ± 0.287	0.170
CI		1.400 ± 0.101	2.086 ± 0.589	<0.001
HI		1.032 ± 0.006	1.0344 ± 0.104	0.311
Coverage(%)		99.501 ± 0.423	99.488 ± 0.481	0.819
Bladder				
Bladder Vol	265.515 ± 120.05 (cc)			
V50(%)	Gy	25.600 ± 21.092	22.185 ± 19.041	<0.001
V35(%)	Gy	39.305 ± 21.191	34.673 ± 20.866	<0.001
V25(%)	Gy	49.403 ± 20.534	44.937 ± 21.128	<0.001
V15(%)	Gy	60.884 ± 15.169	57.795 ± 17.254	0.001
Rectum				
Rectum Vol	83.914 ± 46.148 (cc)			
V50(%)	Gy	51.301 ± 9.909	41.874 ± 14.421	<0.001
V35(%)	Gy	61.184 ± 6.305	54.156 ± 10.467	<0.001
V25(%)	Gy	65.897 ± 4.271	62.573 ± 7.476	<0.001
V15(%)	Gy	69.475 ± 1.772	68.723 ± 3.157	0.009
R Femur				
V5(%)	Gy	25.830 ± 4.834	39.646 ± 7.438	<0.001
L Femur				
V5(%)	Gy	25.818 ± 5.716	39.723 ± 5.222	<0.001
Penile Bulb				
V95(%)	Gy	45.788 ± 21.246	44.705 ± 21.156	1.00
Bowel				
Bowel Vol	1375.541 ± 1037.756 (cc)			
V5(%)	Gy	2.598 ± 1.746	2.303 ± 1.265	0.323

**TABLE 4:** Dose–volume histogram (DVH) parameters (mean ± standard deviation) for planning target volume (PTV) and organs at risk (OARs) with *p*‐values for comparison of Tomo‐Direct (TD) and hybrid plans

**Parameters**	**Unit**	**Tomo‐Direct**	**Hybrid (45 Gy/25 Gy)**	***p‐*Value**
PTV				
PTV Vol	146.559 ± 51.446 (cc)			
PTV D2%	Gy	71.111 ± 0.334	71.188 ± 0.767	0.004
PTV D98%	Gy	70.276 ± 0.124	70.506 ± 0.394	<0.001
PTV D50%	Gy	70.742 ± 0.283	70.971 ± 0.287	<0.001
CI		1.413 ± 0.138	2.086 ± 0.589	<0.001
HI		1.149 ± 0.602	1.0344 ± 0.104	0.222
Coverage(%)		99.332 ± 0.586	99.488 ± 0.481	0.002
Bladder				
Bladder Vol	265.515 ± 120.05 (cc)			
V50(%)	Gy	26.496 ± 21.191	22.185 ± 19.041	<0.001
V35(%)	Gy	40.219 ± 21.417	34.673 ± 20.866	<0.001
V25(%)	Gy	50.018 ± 20.367	44.937 ± 21.128	<0.001
V15(%)	Gy	59.056 ± 17.823	57.795 ± 17.254	0.013
Rectum				
Rectum Vol	83.914 ± 46.148 (cc)			
V50(%)	Gy	51.170 ± 11.609	41.874 ± 14.421	<0.001
V35(%)	Gy	61.529 ± 6.569	54.156 ± 10.467	<0.001
V25(%)	Gy	65.855 ± 5.326	62.573 ± 7.476	<0.001
V15(%)	Gy	69.081 ± 2.724	68.723 ± 3.157	0.065
R Femur				
V5(%)	Gy	26.180 ± 6.51	39.646 ± 7.438	<0.001
L Femur				
V5(%)	Gy	27.148 ± 7.091	39.723 ± 5.222	<0.001
Penile Bulb				
V95(%)	Gy	45.457 ± 20.924	44.705 ± 21.156	0.788
Bowel				
Bowel Vol	1375.541 ± 1037.756 (cc)			
V5(%)	Gy	2.66 ± 1.983	2.303 ± 1.265	0.201

## 4 DISCUSSION

The prostate is located below the bladder and in front of the rectum and gastrointestinal and genitourinary toxicity may occur in hypo‐fractionated RT applications to prostate cancer.^27^ In prostate RT, toxicity is related to the volume irradiated.^28^ In this study, we obtained hypo‐fractionated RT plans by using the planning options in two different modes of (TH and TD) separately and together with a certain combination in the HDR tomotherapy. We chose lateral gantry angles for TD to reduce the rectum and bladder dose while creating a hybrid combination. While determining the lateral angles, we tried to choose them according to the anatomy of the patients by using our clinical experience. While generating lateral gantry angles, we encountered an increase in femoral head doses. There are few data to explain femoral toxicity when higher doses are given to small volumes of the femoral heads. Generally, tolerance of entire femoral heads was limit to V50 Gy < 5%.^29^ We tried different fraction combinations for the hybrid plans. We have seen that approximately two‐thirds of the ratio of TH and one‐third of the TD hybrid plan provides a significant reduction in the bladder and rectum doses. In this way, we were able to limit V50 Gy < 5% without increasing the femoral head dose too much.

There are different types of cancer studies with clinical and dosimetrically outcomes reported in patients treated with hybrid RT. Balaji et al. showed the best treatment plan combination would be achieved with 70%–80% 3D‐IMRT and 20%–30% arc in the hybrid breast RT.^30^ Similarly, Venjakob et al. determined that the hybrid VMAT technique (combination with 80% 3D‐CRT/20% VMAT) has positive advantages in RT of breast cancer.^31^ The study of Zhao et al. showed that the target dose HI and CI was better and critical organ doses decreased by the hybrid IMRT/VMAT technique compared to other techniques for treatment of nasopharyngeal cancer.^32^ Silva et al. presented the clinical application of the hybrid Rapid Arc in patients with locally advanced lung cancer. Hybrid Rapid Arc plans were created with the combination of static (60%) and Rapid Arc (40%) beams and showed advantages for reducing the OARs dose.^33^ We tried different fraction combinations for the hybrid plans in this study. We conclude that 45 Gy/18 fxs for TH and 25 Gy/10 fxs for TD is the best combination.

The number of studies involving hybrid RT of prostate cancer is limited in the literature. The study of Amaloo et al. compared VMAT plans with hybrid plans in prostate patients.^34^ They determined that hybrid plans yielded fewer rectum and bladder doses in almost all cases. Similarly, in our study, we found statistically significant lower doses in the rectal and bladder in hybrid plans. Penile bulb and bowel doses were lower in hybrid plans too. The study of Robar et al. stated that the HybridArc method provides various advantages in different anatomical regions and increases PTV dose homogeneity for prostate treatment.^35^ In a study examining prostate cancer hybrid IMRT and volumetric modulated arc treatment plans, the 25% IMRT technique increased target dose homogeneity and provided rectum protection.^36^ In the current study, as we used beams containing lateral gantry angles used when trying to lower bladder and rectum doses, the hybrid TH/TD plan CI value was statically significantly higher than TH and TD (*p* < 0.001). Also, all planning methods (TD, TH, and hybrid) provided good coverage (%). There was a significant increase in femoral head V5% dose in hybrid plans compared to TH and TD plans (*p* < 0.001). Similarly, Amaloo et al. determined that there was an increase in the maximum doses of the femoral heads in the hybrid VMAT plans.^34^

There are some studies in the literature comparing TH and TD plans of the tomotherapy system. Davidson et al. compared seven‐angle static IMRT plans with TH plans for prostate patients and found conventional IMRT plans were similar to TH.^37^ In the study of Murai et al., they compared five static gantry angled TD plans with TH plans and found the dose to the rectum in TD plans was significantly higher than TH plans.^20^ In our study, the rectum doses were similar between TH and TD but bladder V50 and V35 doses were found to be statistically significantly lower in TH plans compared to TD (*p* < 0.001).

There are several limitations of our study. First of all, we used a small number of patient dosimetric data. The other limitation of this study was similar tomotherapy plans were obtained with fixed gantry angle, iterations, and optimization criteria. Considering that each patient has different PTV, OARs, and anatomical deviations, a specific treatment plan could not be obtained for each patient. Besides, creating and evaluating hybrid plans can cause extra workload for clinics (planning time, delivery quality assurance, etc.). In addition, more research is required on the clinical outcomes of LPC patients treated with hybrid TH/TD. In order to overcome these limitations, large‐scale clinical studies can be carried out with a large number of patients and special plans can be made for each patient. Clinics can start the application with the determined standard data without extra workload.

## 5 CONCLUSION

In this study, we presented a new hybrid delivery method for LPC in HDA tomotherapy. The use of lateral beams in the TH in the hybrid model was a unique approach. Our study showed the advantages of reducing bladder and rectal doses of the hybrid TH/TD method compared to the TH and TD methods. This study may shed light on studies aimed at reducing critical organ dose in hypo‐fractionated prostate RT, which is expected to become widespread in the future.

## CONFLICT OF INTEREST

The authors declare that they have no conflict of interest.

## AUTHOR CONTRIBUTIONS

*Conception and design*: Sibel Karaca; *Administrative support*: Aylin Fidan Korcum Şahin; *Provision of study materials or patients*: Sibel Karaca, Timur Koca, and İsmail Hakkı Sarpün; *Collection and assembly of data*: Sibel Karaca; *Data analysis and interpretation*: Sibel Karaca, Nina Tunçel, and Timur Koca; *Manuscript writing*: Sibel Karaca; *Final approval of manuscript*: All authors.

## ETHICAL APPROVAL

Our study was approved by the Akdeniz University Faculty of Medicine Ethics Committee of Clinical Trials (2020 – KAEK‐813). The study was a retrospective review so subject informed consent was not obtained.
